# Intergenerational family leisure in the COVID-19 pandemic: Some potentials, pitfalls, and paradoxes

**DOI:** 10.1093/geroni/igab046.2903

**Published:** 2021-12-17

**Authors:** Tia Rogers-Jarrell, Deanna Vervaecke, Brad Meisner

**Affiliations:** York University, Toronto, Ontario, Canada

## Abstract

COVID-19 has significantly changed the way we engage in leisure. The influence of public health measures and messaging on leisure put older and younger people alike at increased risk of stress, anxiety, loneliness, and isolation. Despite these similar experiences, ageism and tensions between generations intensified during the pandemic. Thus, it is imperative to encourage strategies that foster connections and solidarity between generations, such as participating in intergenerational family leisure. Intergenerational family leisure can both attenuate negative outcomes heightened or created by the pandemic (i.e., risk reduction) and increase positive experiences (i.e., wellness promotion). However, it is important to recognize that intergenerational family leisure may not be available, or ideal, for everyone, especially during the pandemic. There are longstanding and pandemic-specific pitfalls to engaging in intergenerational family leisure that need to be considered. Further, the conditions and handling of the COVID-19 pandemic have complicated family leisure in paradoxical ways. Many contradictions emerge as we navigate social systems and personal experiences when engaging in intergenerational family leisure during the pandemic. This paper critically presents some of the potentials, pitfalls, and paradoxes associated with connecting multiple generations in and through family leisure during the pandemic.

